# High-grade symptomatic and asymptomatic carotid stenosis in the very elderly. A challenge for proponents of carotid angioplasty and stenting

**DOI:** 10.1186/1471-2261-6-12

**Published:** 2006-03-30

**Authors:** Enzo Ballotta, Giuseppe Da Giau, Carmelo Militello, Bruno Barbon, Aldo De Rossi, Giorgio Meneghetti, Claudio Baracchini

**Affiliations:** 1 Department of Surgical & Gastroenterological Sciences, University of Padua, School of Medicine, Padova, Italy; 2 Department of Neurological Sciences, University of Padua, School of Medicine, Padova, Italy

## Abstract

**Background:**

Carotid angioplasty and stenting (CAS) is often considered as the preferred treatment for severe carotid occlusive disease in patients labelled as "high risk", including those aged 80 or more. We analyzed 30-day stroke risk and death rates after carotid endarterectomy (CEA) for severe symptomatic or asymptomatic carotid disease in patients aged 80 or more, by comparison with the outcome of CAS reported in the recently- published literature.

**Methods:**

A retrospective review was conducted on a prospectively compiled computerized database of all primary CEAs performed by a single surgeon at our institution from 1990 to 2003. Descriptive demographic data, risk factors, surgical details, perioperative strokes and deaths, and other complications were recorded.

**Results:**

In all, 1260 CEAs were performed in 1099 patients; 1145 were performed in 987 patients less than 80 years old, and 115 were performed in 112 patients aged 80 or more. There were 11 perioperative strokes in the 1145 procedures in the younger group, for a stroke rate of 0.8%, and no strokes in the 115 procedures in the older group. The death rates were 0% for the octogenarians and 0.3% for the younger group.

**Conclusion:**

The conviction that older age means higher risk needs to be revised. Patients aged 80 or more can undergo CEA with no more perioperative risks than younger patients. Proponents of CAS should bear this in mind before recommending CAS as the best therapeutic option for such patients.

## Background

Carotid endarterectomy (CEA) has been established as the gold standard for the treatment of both symptomatic and asymptomatic high-grade (≥ 70%) internal carotid artery (ICA) disease, based on the results of several prospective, randomized controlled trials [1-5]. However, though elderly people are typically seen in everyday clinical practice, an increasingly important group of patients, i.e. those aged ≥ 80 years, was rarely recruited [1,2] or excluded from the major surgical trials [3,6]. Due to concern about the excessive risk of complications deriving from concomitant disease or limited post-procedural life expectancy, these patients were labelled as "high risk" and diverted to medical treatment or a seemingly less invasive procedure, such as carotid angioplasty and stenting (CAS). Wherever it was evaluated, best medical care proved less effective than CEA [1-3,7], however, and the results of CAS in elderly patients were hardly encouraging [8-10]. In fact, though many single-centers studies reported that CAS can be performed with acceptable periprocedural complication rates [11], advanced age was considered a significant predictor of periprocedural neurological events after unprotected and protected CAS procedures in several recently-published large single- and multicenter trials [8-10]. This finding is odd, but the authors did not attempt to explain why the outcome of CAS should be significantly worse in older than in younger patients. Indeed, if older age really does mean a higher risk, one would expect the less invasive procedure to be better tolerated by elderly patients and produce better results than CEA. Although reanalyzing outcome in the multicenter surgical trials on the basis of age revealed that patients aged ≥ 75 years benefited more from CEA than younger patients [12] (probably because elderly patients have a greater risk of ischemic stroke on medical treatment and a lower surgical risk), from the trial data it is impossible to say whether this also applies to over 80-year-olds.

The aim of this study was to analyze the perioperative (30-day) stroke risk and death rates of CEA in patients aged ≥ 80 years, as compared with the outcome after CAS reported in the recently-published literature.

## Methods

A retrospective review was conducted on a prospectively compiled computerized database of all primary CEAs performed by a single surgeon at our institution from 1990 to 2003 in symptomatic and asymptomatic patients with high-grade ICA lesions according to the recommendations of the North American Symptomatic Carotid Endarterectomy Trial (NASCET) [1] and the Asymptomatic Carotid Atherosclerosis Study (ACAS) [3]. Patients scheduled for CEA with concomitant coronary artery bypass grafting, or with associated supra-aortic trunk lesions requiring concurrent surgery and patients requiring carotid surgery for recurrent disease were ruled out.

The patients' demographic data were collected, including any history of diabetes mellitus, cigarette smoking, hypertension and coronary artery disease (CAD), other clinical variables, indications for surgery, details of the operation and hospital stay.

The ICA lesion was diagnosed on preoperative traditional digital subtraction angiography (DSA) during the earlier part of this experience (with declining frequency over the years), while duplex ultrasonography scan (DUS) was the only preoperative ICA imaging study performed in most patients from mid-1998 onwards, combined in selected patients with either magnetic resonance (MR) angiography, computed tomography (CT) angiography, or traditional arteriography. The radiologist's estimate of any carotid bulb or ICA stenosis in the final DSA report was recorded using the NASCET method [1]. If no DSA was performed, stenosis was estimated from the findings at preoperative DUS, performed in our vascular laboratory. The velocity criteria used to classify the degree of stenosis revealed a satisfactory correlation with the DSA findings when the degree of stenosis was calculated as the percentage of diameter reduction in compliance with the NASCET method [1]. These criteria have been published elsewhere [13,14], and are validated yearly. Clinical presentation was always classified by the consultant neurologist as transient ischemic attack (TIA; i.e., temporary hemispheric symptoms lasting no more than 24 hours, with complete recovery), amaurosis fugax (transient monocular visual loss), or stroke (neurological deficit persisting more than 24 hours, regardless of the mechanism and related to either cerebral hemisphere). Patients who had non-hemispheric symptoms, such as dizzy spells or vertigo, were included in the asymptomatic group. Preoperative cerebral CT was performed in all symptomatic patients. Preoperative cardiac work-up was tailored to each patient, on the basis of history, electrocardiographic (ECG) findings, and symptoms. Patients with evidence of clinically important CAD underwent echocardiography or dipyridamole-thallium stress tests followed by coronary arteriography as indicated. Pre- and postoperative cranial nerve assessment was done in all patients by a neurologist and an otolaryngologist. Vocal cord movements were assessed by direct fiberoptic laryngoscopy in patients presenting symptoms and/or signs of vagus nerve injury [15]. Preoperative patient preparation was standardized. To reduce the incidence of neck hematoma, antiplatelet therapy (aspirin or dipyridamole, and ticlopidine or clopidogrel in the final period) was suspended at least 1 week before the operation, and was not resumed until the patient was discharged from the hospital.

All CEA procedures involved either traditional CEA with patching (n = 302) or eversion CEA (n = 958). The technical details of both procedures have been described elsewhere [16,17]. All CEAs were performed with patients under deep general anaesthesia and cerebral protection involving continuous perioperative electroencephalographic monitoring (EEG) for selective shunting. All perioperative EEGs were visually analyzed by a neurologist with an extensive experience in the interpretation of studies during sleep, either natural or induced by hypnotic or anaesthetic agents. Shunting criteria were based exclusively on EEG changes consistent with cerebral ischemia. Completion imaging studies were never performed.

Patients were usually monitored in the recovery room for 2 hours until their blood pressure and neurological status were considered acceptable before being transferred to a regular nursing unit specializing in vascular care and monitored for the next 12 to 24 hours after surgery. All patients with severe headache were observed for hyperperfusion syndrome, and hypertension was treated aggressively. Most patients were discharged 48–72 hours after CEA.

The endpoints of this study were perioperative stroke and death, and cardiac complications, which were prospectively recorded according to the guidelines of the Ad Hoc Committee on Reporting Standards for Cerebrovascular Disease, Society for Vascular Surgery/North American Chapter of the International Society of Cardiovascular Surgery [18]. *Minor stroke *was defined as a focal neurological deficit of acute onset, lasting more than 24 hours and not leading to disability. *Major stroke *was defined as a focal neurological deficit leading to disability and permanent handicap.

All patients were assessed postoperatively by a consultant neurologist at the awakening from the anaesthesia and before the discharge. After discharge, visiting nurses monitored the patients' blood pressure and neurological status. Clinical evaluation and DUS were performed systematically by a consultant neurologist and two experienced technologists in all surviving patients at 1, 6 and 12 months, and once every postoperative year thereafter, assessing any residual ICA stenosis, angulation, recurrent ICA disease, or occlusion with an Acuson Sequoia 512 ultrasound system (Mountain View, Calif). Cerebral CT or MR scans were performed in all patients presenting a new neurological deficit.

Cardiac complications included: 1) myocardial infarction (MI), with a diagnosis made on the basis of creatine kinase enzyme levels and ECG findings; 2) pulmonary edema confirmed by the official reading of the chest radiograph; 3) documented ventricular fibrillation or primary cardiac arrest; and 4) new complete heart block requiring a pacemaker. A postoperative ECG was routinely obtained in all patients who had a history of CAD, CHF, or arrhythmia (rhythm other than sinus). Cardiac isoenzymes were obtained in all patients who had new findings on the postoperative ECG.

### Data analysis

All statistical analyses were performed with the SPSS 10.0 statistical software package (SPSS, Chicago, Ill). Univariate analysis was performed on all clinical, morphological, and procedural variables, with Student's *t *test (two-tailed) for continuous variables, and chi-square analysis (two-tailed) for categorical variables. Statistical significance was inferred for p < .05. Since each perioperative outcome was correlated with the surgical procedure, and since patients who underwent bilateral CEAs were exposed to twice the risk of stroke or death, several data items were analyzed vis-a-vis surgical procedures rather than patients.

## Results

Overall, 1260 CEAs were performed in 1099 patients, 112 of them were aged ≥ 80 years (10.2%; 129 CEAs), while the other 987 patients (89.8%; 1131 CEAs) were younger. The preoperative demographic data for the two groups considered are shown in Table 1. The mean age was 84.2 years (range, 80 to 93) in the older group and 67.6 years (range, 31 to 79) in the younger group. The incidence of arterial hypertension was statistically higher in the older group (70.5% vs 58.2%, p = .002), while older patients were significantly less likely to have diabetes mellitus (19.6% vs 30.1%, p = .01). There was no statistically significant difference between the two groups in current or past history of smoking, CAD, and indications for surgery. The older patients experienced a statistically higher rate of perioperative EEG changes suggestive of cerebral ischemia requiring shunting (45.2% vs 17.6%, p < .001), so they needed shunting statistically more often the younger group (35.6% vs 11.5%, P < .001) (Table 1). The type of CEA procedure used (traditional with patching or eversion) was much the same in the two groups, but eversion CEA was performed more often than traditional CEA, both in the series as a whole (76% vs 24%, P < .001) and in each group (69.5% vs 30.5% in the older group, p = <. 001; and 76.7% vs 23.3% in the younger group, p < .001) (Table 1).

**Table 1:** Baseline characteristics, and anatomical/technical data

	**Patients ≥ 80 yrs (n = 112)**	**Patients < 80 yrs (n = 987)**	***p *value**
CEAs, n	115	1145	
Mean age, yrs (range)	84.2 (80–93)	67.6(31–79)	
Male, n(%)	69(61.6)	672(68.1)	.17
			
Risk factors			
Hypertension, n(%)	79 (70.5)	575 (58.2)	.01
Current smoking or past history of smoking, n(%)	86 (76.8)	697 (70.6)	.19
Diabetes mellitus, n(%)	22 (19.6)	331 (30.1)	.002
CAD, n(%)	52 (46.4)	446(45.1)	.84
			
Symptomatic carotid disease, n(%)	76(66.1)	763 (66.7)	.92
Asymptomatic carotid disease, n(%)	39 (33.9)	382 (33.3)	.92
			
Contralateral carotid occlusion, n(%)	59 (52.6)	94 (9.5)	<.001
CEA with patching, n(%)	35 (30.5)	267 (23.3)	.11
Eversion CEA, n(%)	80 (69.5)	878 (76.7)	.11
Intraoperative EEG changes, n(%)	52 (45.2)	202 (17.6)	<.001
Shunting, n(%)	41 (35.6)	132(11.5)	<.001

CEA, carotid endarterectomy; TIA, transient ischemic attack; CAD, coronary artery disease; EEG, electroencephalographic.

### Perioperative mortality and stroke rates

Overall, the perioperative mortality rate was 0.2% (3 of 1260), and the stroke rate was 0.9% (11 of 1260) with a combined mortality and stroke rate of 1.1% (Table 2). No significant differences emerged between the two groups in terms of perioperative neurological events or death. Perioperative death and stroke occurred only in the younger group. There were 3 perioperative deaths: 2 due to MI, and 1 to stroke. The only fatal perioperative stroke occurred in a patient with symptomatic disease undergoing CEA with patching to treat a severe ulcerated left ICA lesion. All remaining strokes (6 CEA procedures with patching, 4 eversion CEAs) occurred in symptomatic patients, and all but two were major in severity. In all cases stroke occurred within the first 24 hours of surgery, while the patient was in the recovery room: DUS immediately confirmed an ICA occlusion in the patched patients, whereas it demonstrated ICA patency in the everted patients. Among the patched patients, 4 underwent re-operation consisting of a thrombectomy and new patch-plasty: there was some improvement in the neurological status of only one patient and none in the others. The remaining two strokes involved the hemisphere contralateral to the operated side (one of these was ipsilateral to an occluded ICA). Among the 4 strokes in everted patients, two were major and two minor. Both major strokes occurred in patients (one of them was shunted) with a mildly diseased contralateral ICA and were probably embolic (from the aortic arch or from the heart), because cerebral CT scans demonstrated a cortical infarction in the territory of the middle cerebral artery. Both minor strokes were most likely hemodynamic in nature, as suggested by the CT images: one developed in the hemisphere contralateral to the revascularized ICA and ipsilateral to an occluded ICA.

**Table 2:** Perioperative (30-day) results

	**Total (1099 pts, 1260 CEAs)**	**Patients ≥ 80 yrs (n = 112, 115 CEAs)**	**Patients < 80 yrs (n = 987, 1145 CEAs)**	**p value**
Stroke, n(%)	11(0.9)	0	11(0.8)	.61
major	9		9	
minor	2		2	
Death, n(%)	3 (0.2)	0	3 (0.3)	1
stroke-related	1			
				
Cardiac complications, n(%)	11(0.9)	1 (0.9)	10 (0.9)	1
fatal	2		2	
non-fatal	9 (0.7)		8 (0.7)	
				
Nerve injuries, n(%)	64(5)	4 (3.4)	60 (5.2)	.51

### Other complications

Overall, there were 11 perioperative cardiac complications (0.9%)(Table 2). The only two fatal MIs occurred in two younger symptomatic patients. Nine patients (0.7%), one in the older group and 8 in the younger group, had perioperative congestive heart failure (CHF), including four patients with a history of CHF. Moreover, one of the patients with CHF also had a postoperative nonfatal MI. The incidence of CHF or MI did not statistically differ between older and younger groups (Table 2). No hyperperfusion syndrome was observed in any of the patients.

Other important surgical morbidities included an overall 5% incidence of nerve injury (64 of 1260). The cranial nerve and the cervical nerve injuries amounted to 4.2% (54 of 1260) and 0.8% (10 of 1260), respectively: there were 31 hypoglossal nerve injuries, 12 recurrent laryngeal nerve injuries, 7 superior laryngeal nerve injuries, 4 marginal mandibular nerve injuries, 7 greater auricular nerve injuries and 3 transverse cervical nerve injuries. All nerve dysfunctions were transient, and all but 4 recurrent laryngeal nerves recovered completely within 6 months of CEA; 2 patients took 12 months to recover and 2 did not recover until 31 and 37 months later. There was no statistically significant difference between the groups (Table 2).

### Comparison of perioperative outcome on CEA and CAS

Comparisons were drawn between the incidence of perioperative stroke and death after CEA in patients in this study aged ≥ 80 years and the representative results from three recently-published series dealing specifically with CAS in elderly patients [9,10,19], as given in Table 3. The outcome was better after CEA than after CAS in all-patient categories, but the most impressive difference was in the elderly patients.

**Table 3:** Periprocedural (30-day) stroke and death incidence after carotid angioplasty and stenting by patient age

**Author**	**Patients ≥ 80 yrs ** **Stroke and death, n(%)**	**Patients < 80 yrs** ** Stroke and death, n(%)**
Roubin ^9^	12/63 (16)	31/465 (6)
Hobson ^10^	6/53(11.3)	21/650 (3.2)
Kastrup ^19^	12/99(12.1)	N/A
		
Total	30/215 (13.9)	52/1115(4.7)

N/A = not available.

## Discussion

The results of this study show that CEA for symptomatic and asymptomatic high-grade carotid disease can be performed safely in patients aged ≥ 80 years, with perioperative stroke risk and death rates comparable with those of younger patients. This finding is consistent and extends the results of our two previous studies on early and long-term outcomes in patients aged 75 or more [20] and in over 80 year-olds with contralateral carotid occlusion [21].

Older patients were significantly less likely than younger patients to have a history of diabetes mellitus and this could represent a selection bias, with only healthier older patients being referred for CEA. Though we cannot infer this from the current data, we do not believe this is the case. Even if this was, the significantly higher incidence of arterial hypertension in the older group should counterbalance any influence of this bias. The two groups had similar indications for surgery, but older patients had a statistically higher rate of intraoperative EEG changes suggesting cerebral ischemia and needed shunting statistically more often than younger patients. This may be owing to the significantly greater presence of contralateral ICA occlusion in elderly patients, making the cerebral collateral flow insufficient to withstand the stress of clamping [22]. Shunting did not appear to have a negative effect on perioperative outcome, however.

Although there has been concern about the safety and effectiveness of CEA in patients aged ≥ 80 years, given their higher risk of complications due to associated comorbidities and their limited life expectancy, numerous series have reported excellent results in this patient population [23-29], demonstrating that the very elderly could benefit from CEA even more than others (Table 4).

**Table 4:** Recent series reporting perioperative outcome for carotid endarterectomy in elderly patients

Author	Patients ≥ 80 yrs		Patients < 80 yrs	
	Stroke, n(%)	Death, n (%)	Stroke,(n %)	Death, n(%)
Schultz ^23^	1/116(0.9)	1/95(1)	1/105 (0.9)	2/90 (2.2)
Treiman ^24^	4/183 (2.2)	3/146(2)	47/1487(3.2)	12/1176(1)
Perler ^25^	13/1036(1.2)	14/1036(1.3)	153/8882(1.7)	76/8882(0.8)
Maxwell ^26^	7/218 (3.2)	2/187 (1)	62/2180(1.4)	31/1783(1.7)
Oszvath ^27^	1/125 (0.8)	0	33/3932 (0.8)	0
Rockman ^28^	3/161 (1.9)	0	17/537 (3.2)	2/537 (0.4)
Miller ^29^	4/360(1.1)	7/360(1.9)	14/1857(0.8)	15/1857(0.8)
				
Total	33/2199(1.5)	27/2110(1.3)	327/18980(1.7)	138/18257(0.7)

The suggestion that patients aged ≥ 80 years are good candidates for CAS procedures and that CAS is consequently preferable to CEA in this patient population appears to be based on two premises that remain to be seen, i.e. that CEA is a high risk procedure and that CAS is safer than CEA. The first premise is not supported by the perioperative outcome emerging from our own and many other institutional series [23-29]. The second is belied by the analysis of the periprocedural results of the three series considered [8-10], which revealed an alarming, unacceptably high non-fatal stroke and death rate among the very elderly, ranging from 11.3% of Hobson's series [10] to 16 % of Roubin's series [9], with an overall perioperative stroke risk and death incidence of 13.9% when the outcomes of the three series are pooled [8-10]. The conclusion reached by all three studies was that octogenarians should be considered high-risk patients for CAS procedures and the common practice of recommending CAS for older patients was questionable and should be abandoned until the results of controlled clinical trials become available.

### Limitations of the study

Our study has several drawbacks. Though our data were collected prospectively, the analysis is retrospective in nature. Any major and minor perioperative stroke is unlikely to have been overlooked in our study because all patients were pre- and postoperatively evaluated by two board-certified neurologists. The study would have benefited from a comparison of perioperative stroke and death incidence on symptomatic and asymptomatic patients aged ≥ 80 years with high-grade carotid stenoses who were followed up without surgery. Because this study was retrospective, we could not (nor did we intend to) analyze data for patients with indications for CEA who did not undergo surgery. Moreover, we have no way to knowing whether our elderly patients are really comparable with the elderly populations in the CAS series. Unlike our series, the CAS studies included many recurrent carotid diseases, while they excluded patients with long subocclusive carotid lesions, whereas many of our patients had surgery for a "string sign" lesion. Finally, though our institutional outcomes, and those of the CAS series considered here, may not be representative of more generalized experiences with either technique, so attempting to draw definitive conclusions from the comparison would be unjustifiable, a non-fatal stroke and death rate of 13.9% would be unacceptable in any patient population.

## Conclusion

The conviction that older age means higher risk needs to be revised. Patients aged ≥ 80 years can undergo CEA with no greater perioperative risks than younger patients. Those advocating CAS in "high risk" patients should bear this in mind, as well as the fact that periprocedural risk of stroke and death after CAS increases with age. In the light of our findings, and in good agreement with the low perioperative complication rates of many institutional series, CEA remains the best therapeutic option in elderly patients with high-grade symptomatic and asymptomatic carotid stenosis.

## Abbreviations

**CEA **= carotid endarterectomy; **ICA **= internal carotid artery; **CAS **= Carotid Angioplasty and Stenting; **NASCET **= North American Symptomatic Carotid Endarterectomy Trial; **ACAS **= asymptomatic carotid atherosclerosis study; **CAD **= coronary artery disease; **DSA **= digital subtraction angiography; **DUS **= duplex ultrasonography scan; **MR **= magnetic resonance; **CT **= computed tomography; **TIA **= transient ischemic attack; **ECG **= electrocardiogram; **EEG **= electroencephalography; **MI **= myocardial infarction; **CHF **= congestive heart failure

## Competing interests

The author(s) declare that they have no competing interests.

## Authors' contributions

**EB **conceived of the study, designed major parts of the study, performed all surgical procedures, coordinated the study, participated in the statistical analyses and drafted the manuscript; **GDG **helped coordinate the study and helped draft the manuscript; CM contributed to literature search and helped draft the manuscript; **BB **collected data and helped draft the manuscript; **ADR **participated in the statistical analyses and drafted the manuscript; **GM **contributed to the pre- and postoperative neurological evaluation of all patients and helped to draft the manuscript; **CB **contributed to the pre- and postoperative neurological evaluation of all patients and helped to draft the manuscript.

## Pre-publication history

The pre-publication history for this paper can be accessed here:



## References

[B1] North American Symptomatic Carotid Endarterectomy Trial Collaborators (1991). Beneficial effect of carotid endarterectomy in symptomatic patients with high-grade carotid stenosis. N Engl J Med.

[B2] European Carotid Surgery Trialists Collaborative Group (1998). Randomized trial of endarterectomy for recently symptomatic MRC Europe carotid stenosis: final results of the MRC European Carotid Surgery Trial. Lancet.

[B3] Executive Committee for the Asymptomatic Carotid Atherosclerosis Study (1995). Endarterectomy for asymptomatic carotid artery stenosis. JAMA.

[B4] Mayberg MR, Wilson SE, Yatsu F, Weiss DG, Messina L, Hershey LA, Colling C, Eskridge J, Deykin D, Winn HR (1991). Carotid endarterectomy and prevention of cerebral ischemia in symptomatic carotid stenosis. Veterans Affairs Cooperative Studies Program 309 Trialist Group. JAMA.

[B5] Hobson RW, Weiss DG, Fields WS, Goldstone J, Moore WS, Towne JB, Wright CB (1993). Efficacy of carotid endarterectomy for asymptomatic carotid stenosis. N Engl J Med.

[B6] Rothwell PM (2001). Carotid endarterectomy and prevention of stroke in the very elderly. Lancet.

[B7] Barnett HJM, Taylor DH, Eliasziw M, Fox AJ, Ferguson GG, Haynes RB, Rankin RN, Clagett GP, Hachinski VC, Sackett DL, Thorpe KE, Meldrum HE, Spence JD (1998). Beneficial effect of carotid endarterectomy in patients with symptomatic moderate or severe carotid stenosis. North American Symptomatic Carotid Endarterectomy Trial Collaborators. N Engl J Med.

[B8] Chastain HD, Gomez CR, Iyer SS, Roubin GS, Vitek JJ, Terry JB, Levine RL (1999). Influence of age upon complications of carotid artery stenting. UAB Neurovascular Angioplasty Team. J Endovasc Surg.

[B9] Roubin GS, New G, Iyer SS, Vitek JJ, Al-Mubarak N, Liu MW, Yadav J, Gomez G, Kuntz RE (2001). Immediate and late clinical outcome of carotid artery stenting in patients with symptomatic and asymptomatic carotid artery stenosis. Circulation.

[B10] Hobson RW, Howard VJ, Roubin GS, Brott TG, Ferguson RD, Popma JJ, Graham DL, Howard G, CREST investigators (2004). Carotid artery stenting is associated with increased complications in octogenarians: 30-day stroke and death rates in the CREST lead-in phase. J Vasc Surg.

[B11] Kastrup A, Groschel K, Krapf H, Brehm BR, Dichgans J, Schulz JB (2003). Early outcome of carotid angioplasty and stenting with and without cerebral protection devices: a systematic review of the literature. Stroke.

[B12] Alamowitch S, Eliasziw M, Algra A, Meldrum H, Barnett HJM (2001). Risk, causes and prevention of ischemic stroke in elderly patients with symptomatic internal-carotid- artery stenosis. Lancet.

[B13] Ballotta E, Da Giau G, Abbruzzese E, Saladini M, Renon L, Scannapieco G, Meneghetti G (1999). Carotid endarterectomy without angiography: can clinical evaluation and duplex ultrasonographic scanning alone replace traditional arteriography for carotid surgery work-up? A prospective study. Surgery.

[B14] Ballotta E, Da Giau G, Renon L, Abbruzzese E, Saladini M, Moscardo P, Baracchini C, Meneghetti G (1999). Symptomatic and asymptomatic carotid artery lesions in peripheral vascular disease: a prospective study. Int J Surg Investig.

[B15] Ballotta E, Da Giau G, Renon L, Name S, Saladini M, Abbruzzese E, Meneghetti G (1999). Cranial and cervical nerve injuries after carotid endarterectomy: a prospective study. Surgery.

[B16] Ballotta E, Da Giau G, Saladini M, Abbruzzese E, Renon L, Toniato A (1999). Carotid endarterectomy with patch closure versus carotid eversion endarterectomy and reimplantation: a prospective randomized study. Surgery.

[B17] Ballotta E, Da Giau G, Piccoli A, Baracchini C (2004). Durability of carotid endarterectomy for treatment of symptomatic and asymptomatic stenoses. J Vasc Surg.

[B18] Baker JD, Rutherford RB, Bernstein EF, Courbier R, Ernst CB, Kempczinski RF, Riles TS, Zarins CK (1988). Suggested standards for reports dealing with cerebrovascular disease. J Vasc Surg.

[B19] Kastrup A, Schulz JB, Raygrotzki S, Groschel K, Ernemann U (2004). Comparison of angioplasty and stenting with cerebral protection versus endarterectomy for treatment of internal carotid artery stenosis in elderly patients. J Vasc Surg.

[B20] Ballotta E, Da Giau G, Saladini M, Abbruzzese E (1999). Carotid endarterectomy in symptomatic and asymptomatic patients aged 75 years or more: Perioperative mortality and stroke risk rate. Ann Vasc Surg.

[B21] Ballotta E, Renon L, Da Giau G, Barbon B, Terranova O, Baracchini C (2004). Octogenarian with contralateral carotid artery occlusion: a cohort at higher risk for carotid endarterectomy?. J Vasc Surg.

[B22] Baker WH, Howard VJ, Howard G, Toole JF, for the ACAS Investigators (2000). Effect of contralateral occlusion on long-term efficacy of endarterectomy in the Asymptomatic Carotid Atherosclerosis Study (ACAS). Stroke.

[B23] Schultz RD, Sterpetti AV, Feldhaus RJ (1988). Carotid endarterectomy in octogenarians and nonagenarians. Surg Gynecol Obstet.

[B24] Treiman RL, Wagner WH, Foran RF, Cossman DV, Levin PM, Cohen JL, Treiman GS (1992). Carotid endarterectomy in the elderly. Ann Vasc Surg.

[B25] Perler BA, Dardik A, Burleyson GP, Gordon TA, Williams GM (1998). Influence of age and hospital volume on the results of carotid endarterectomy: a statewide analysis of 9918 cases. J Vasc Surg.

[B26] Maxwell JG, Taylor AJ, Maxwell BG, Brinker CC, Covington DL, Tinsley E (2000). Carotid endarterectomy in the community hospital in patients age 80 and older. Ann Surg.

[B27] Ozsvath KJ, Darling RC, Tabatabai L, Roddy SP, Paty PS, Chang BB, Kreinberg PB, Shah DM (2002). Carotid endarterectomy in the elderly: does gender effect outcome?. Cardiovasc Surg.

[B28] Rockman CB, Jacobowitz GR, Adelman MA, Lamparello PJ, Gagne PJ, Landis R, Riles TS (2003). The benefits of carotid endarterectomy in the octogenarian: a challenge to the results of carotid angioplasty and stenting. Ann Vasc Surg.

[B29] Miller MT, Comerota AJ, Tzilinis A, Daoud Y, Hammerling J (2005). Carotid endarterectomy in octogenarians: does increased age indicate "high risk"?. J Vasc Surg.

